# The Role of Immediate Recurrent Laryngeal Nerve Reconstruction for Thyroid Cancer Surgery

**DOI:** 10.1155/2010/846235

**Published:** 2010-06-14

**Authors:** Tetsuji Sanuki, Eiji Yumoto, Ryosei Minoda, Narihiro Kodama

**Affiliations:** Department of Otolaryngology-Head and Neck Surgery, Graduate School of Medicine, Kumamoto University, 1-1-1 Honjo, Kumamoto 860-8556, Japan

## Abstract

Unilateral vocal fold paralysis (UVFP) is one of the most serious problems in conducting surgery for thyroid cancer. Different treatments are available for the management of UVFP including intracordal injection, type I thyroplasty, arytenoid adduction, and laryngeal reinnervations. The effects of immediate recurrent laryngeal nerve (RLN) reconstruction during thyroid cancer surgery with or without UVFP before the surgery were evaluated with videostroboscopic, aerodynamic, and perceptual analyses. All subjects experienced postoperative improvements in voice quality. Particularly, aerodynamic analysis showed that the values for all patients entered normal ranges in both patients with and without UVFP before surgery. Immediate RLN reconstruction has the potential to restore a normal or near-normal voice by returning thyroarytenoid muscle tone and bulk seen with vocal fold denervation. Immediate RLN reconstruction is an efficient and effective approach to the management of RLN resection during surgery for thyroid cancer.

## 1. Introduction

Unilateral vocal fold paralysis (UVFP) is one of the most serious problems in the management of thyroid cancer. The vocal folds may be paralyzed at the time of presentation, or the recurrent laryngeal nerve (RLN) may need to be sacrificed even when the RLN is functioning preoperatively. UVFP causes breathy voice, shortening of phonation, and aspiration. The negative impact of UVFP on a patient's quality of life has been confirmed by several outcome measurements [1, 2].

Different treatments are available for the management of UVFP including intracordal injection [3], type I thyroplasty [4], arytenoid adduction [5], and laryngeal reinnervations [6–9]. Laryngeal reinnervation has several advantages over other techniques. It has the potential of restoring a normal or near normal voice. RLN reinnervation can prevent the progressive loss of thyroarytenoid muscle tone and bulk [7, 9, 10] as seen with vocal fold denervation, which can limit the long-term results of the conventional static laryngoplasty procedure. 

We report on cases involving the immediate reconstruction of the RLN during thyroid cancer surgery in patients with or without UVFP preoperatively and voice outcomes following the procedure with videostroboscopic, aerodynamic, and perceptual analyses.

## 2. Materials and Methods

During the period from 2000 to 2008, at Kumamoto University Hospital, Japan, we reconstructed the RLN in 12 patients who had UVFP or whose unilateral RLN needed to be sacrificed due to thyroid cancer (Table 1). In 12 patients with thyroid cancer involving unilateral RLN, we conducted direct anastomosis, free nerve grafting, and ansa cervicalis to RLN anastomosis in 1, 9, and 2 patients, respectively. There were 8 women and 4 men, and the ages at the time of reconstruction ranged from 18 to 82 years (mean 61.8). Six of the 12 patients (50%) had UVFP preoperatively and were classified as group I. The remaining six patients did not have UVFP before surgery. However, their RLNs were sacrificed due to invasion by cancer, and these 6 patients were classified as group II (Table 2).

### 2.1. Method of RLN Reconstructions

**Figure 1:**
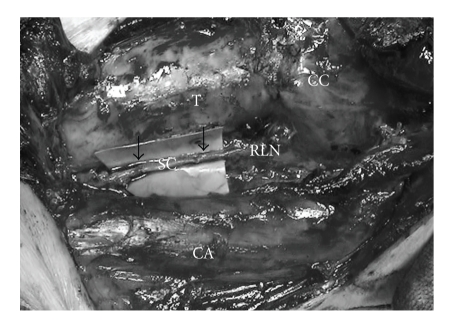
Free nerve graft. Supraclavicular nerves was used to fill the defect (allow). T: trachea, CA: common carotid artery. SC: supraclavicular nerve, CC: cricoid cartilage.

The immediate RLN reconstruction was done at the time of surgery for primary or recurrent thyroid cancer. The ends of the severed RLN were anastomosed directly when possible. When the defect was longer than 5 mm, a free nerve graft taken from the transverse cervical nerve, supraclavicular nerve, or ansa cervicalis was used to fill the defect (Figure 1). When the proximal stumps of the RLN could not be utilized for nerve repair, ansa cervicalis to RLN anastomosis was performed. The ipsilateral ansa cervicalis was identified on the surface of the internal jugular vein, and its branches to the sternohyoid muscles were dissected. The major branch or usually the branch common to these branches was transected, and the proximal end was anastomosed to the distal stump of the RLN. Most commonly, the ipsilateral ansa cervicalis was used for reinnervation. In one case (Patient no. 7) suffering from recurrent disease, due to loss of the ipsilateral ansa cervicalis nerve in the excessive cicatricial tissue, the contralateral ansa cervicalis was used. In Patient no. 2, thyroid cancer has invaded the distal portion of the RLN at the Berry ligament. We resected the RLN at the entrance of the larynx. The inferior pharyngeal constrictor muscle was divided along the lateral edge of the thyroid cartilage in order to find the distal stump of the RLN. The stumps were anastomosized with the supraclavicular nerve. The anastomosis was usually made with three, or sometimes four, stitches of 8–0 or 9–0 nylon thread using microsurgical instruments with an operation microscope or a surgical magnifying glass.

### 2.2. Voice Outcome Measurements

The patients received videostroboscopic, aerodynamic, and perceptual analyses pre- and postoperatively. In group I, some patients were not analyzed preoperatively. The postoperative assessment was done no less than 6 months after operation in all patients.

For videostroboscopic evaluation, each patient performed a sustained phonation of the vowel /e/ or /i/ at his or her habitual pitch and loudness. Images were recorded using a videoendoscopic (VNL-1171; Pentax, Tokyo, Japan) and stroboscopic unit (LS-3A; Nagashima, Tokyo, Japan) onto a digital videocassette recorder (DVCPRO; Panasonic, Yokohama, Japan) to assess the mucosal wave and glottal closure. We rated the mucosal wave of vocal fold vibration and glottal closure using a four-point grading scale (0 = worst, 3 = the best).

For aerodynamic evaluation, each patient was asked to produce a sustained vowel /a/ at a comfortable pitch and loudness for as long as possible. The maximum phonation time (MPT) and mean airflow rate (MFR) were measured using a phonation analyzer (PS-77E, Nagashima, Tokyo, Japan).

Perceptual voice evaluations were conducted using the GRBAS (Overall Grade: G, Roughness: R, Breathiness: B, Asthenia: A, and Strain: S) rating scale [11]. Three of 5 parameters (G, R, and B) were analyzed because UVFP causes breathy dysphonia. S and A were not analyzed due to limited application in the measurement of UVFP. Each parameter was rated by a speech language pathologist according to a 4-point scale (0 = normal; 1 = slight disturbance; 2 = moderate disturbance, 3 = sever disturbance). 

Perceptual analyses were performed following standard procedures in cooperation with a trained speech therapist.

Statistical analyses (SigmaStat 3.5 for windows, San Joe, CA) were performed using the Wilcoxon signed-rank test and unpaired *T* test: *P*-values less than .05 were considered statistically significant.

## 3. Results

The voices of the patients who underwent the immediate RLN reconstruction began to improve after a certain period postoperatively. Table 2 summarize the results of the videostroboscopic, aerodynamic, and perceptual examinations.

The follow-up period in this study (Table 1) ranged from 7 to 103 months (average of 34.6 months). We show the data on the periods of vocal cord paralysis depending on the referral form and or the patients' history in group I.

### 3.1. Videostroboscopic Findings

No visible vocal fold movement was detected during the follow-up period. The postoperative score of mucosal wave (2.5 ± 0.5) in group I was significantly greater than the preoperative score (1.2 ± 1.0). The postoperative glottal closure (2.7 ± 0.5) in group I was significantly greater than the preoperative score (1.5 ± 0.8). Nine of the 12 patients had complete glottal closure during phonation. The small glottal gap remained in 2 patients (Table 2). In one of the 12 patients (Patient no. 10), the vocal folds could not be observed during phonation because of bilateral arytenoidal overhang on the glottis. Despite this, the patient's voice quality was quite good as demonstrated by other measurements. All patients recovered the mucosal wave after the operation with the exception of one patient (Patient no. 9) who also suffered from a polypoid vocal fold.

### 3.2. Aerodynamic Findings

**Figure 2:**
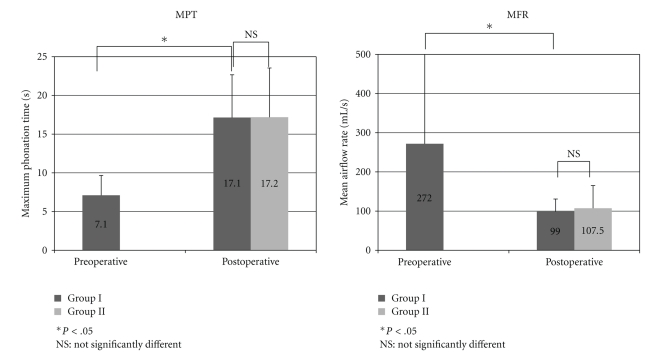


Normal speakers usually have an MPT of more than 10 seconds and an MFR between 100 ml/sec and 200 ml/sec. The postoperative recordings of MPT (16.2 sec ± 6.2) in group I were significantly greater than the preoperative levels (7.1 sec ± 2.55). The postoperative MFR (110.3 ml/sec ± 38.4) in group I was significantly reduced compared with the preoperative levels (271 ml/sec ± 325.1) (Figure 2). In group II, three of the 6 patients did not receive a preoperative examination. Therefore, it was not possible to make a statistical comparison of preoperative and postoperative data. However, the postoperative results of MPT and MFR showed that patients' voices were returned to a normal condition. There were no significant differences between the postoperative data of both groups (Figure 2).

### 3.3. Perceptual Analysis

**Figure 3:**
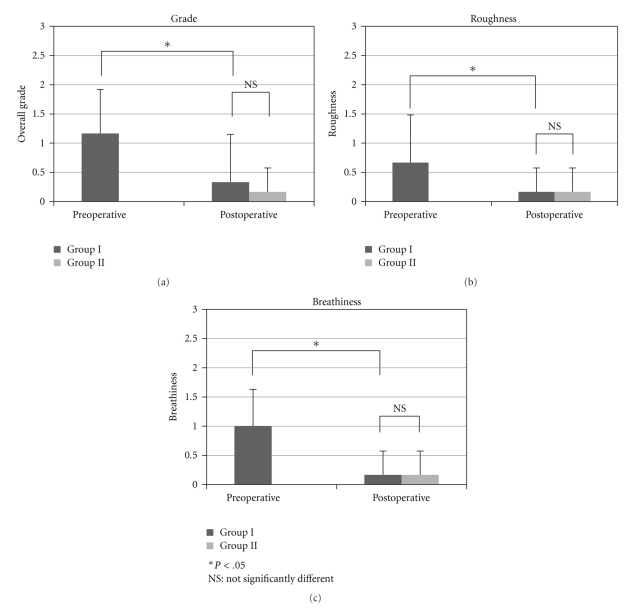


The comparative perceptual analyses of each scale in group I and II are shown in Figure 3.

In group I, the mean score for G was 1.2 ± 0.8 preoperatively and 0.3 ± 0.8 postoperatively. The mean scores for B were 0.7 ± 0.8 preoperatively and 0.2 ± 0.4 postoperatively. The mean score for R was 1.0 ± 0.6 preoperatively and was reduced to 0.2 ± 0.4 postoperatively. All three characteristic scores were significantly reduced postoperatively.

In group II, half of the cases did not receive preoperative examinations. Also, Patient no. 9 had a polypoid vocal cord which caused an elevated score of the perceptual analysis before the surgery. However, the postoperative perceptual scores were within normal ranges.

## 4. Discussion

A major morbidity associated with thyroid surgery is injury to the RLN, resulting in poor voice quality and the potential for recurrent aspiration.

Reconstitution of vocal fold thickness and median position can be accomplished by vocal fold augmentation [3], vocal fold medialization with type I thyroplasty [4], arytenoid adduction [5], or RLN reinnervation [6–8]. These techniques can be used alone or in combination to achieve improved vocal and swallowing functions. RLN reinnervation has several advantages over other techniques. It has the potential of restoring a normal or near-normal voice by returning thyroarytenoid muscle tone and bulk [6, 7, 9, 12, 13] in contrast with the conventional laryngoplasty procedure [7]. Reconstruction of the RLN in the management of UVFP during the thyroid cancer surgery is rarely reported [9, 14–18]. Some disadvantages of this technique are the requirement of a delayed time to voice improvement, the requirement of intact donor and recipient nerves, and the possible delay or failure of reinnervation in elderly patients.

In this study, we assessed the outcome of immediate RLN reconstruction during thyroid cancer surgery with or without UVFP before the surgery. In particular, the aerodynamic analysis showed that all measured values entered normal ranges in both groups I and II. It should be noted that as patients in group II had a normal voice preoperatively, phonatory function was not analyzed in some cases. As such, aerodynamic analyses were employed to measure changes in phonatory function postoperatively. These results coincided with the glottal closure and mucosal wave assessed with videostroboscopy (Table 2). These results were achieved with no bias as to the method of RLN reconstruction such as direct anastomosis, nerve grafting, and ansa cervicalis and RLN anastomosis. Nerve anastomosis under microscopic control was important for precise neuronal repairs.

Several techniques of peripheral nerve repair exist, including microsuturing, gluing [19, 20] and grafting [21]. Cyanoacrylate synthetic glue has therefore been proposed because its application for nerve repair is relatively easy, it maintains the anastomosis even while under tension, and it avoids all risk of viral transmission [22, 23]. In spite of this, this adhesive has received criticism due to its toxicity, excessively slow resorption, as well as the possibility of the induction of an inflammatory reaction in the perineural tissues [24, 25]. In the present study, the standard microsuturing technique was employed for RLN reconstruction. RLN reconstruction requires surgical precision; however, most surgeons involved in the treatment of thyroid cancer and RLN should possess the requisite proficiency to conduct the procedure.

 In group I, all patients who had UVFP with vocal fold atrophy before the surgery experienced restored phonatory function after the immediate RLN reconstruction during thyroid cancer surgery. According to other studies, clinical improvements have been noted 2–4 months after the reinnervation [10, 15, 16, 26], which corresponds with our findings of 3-4 months (not shown). Green et al. [7] demonstrated in a canine model that at 5-6 months, evoked electromyography (EMG) indicated some degree of reinnervation. These EMG findings in animal experiments were also reported in clinical patients who underwent serial EMG studies after laryngeal reinnervation for UVFP [13, 26]. Miyauchi et al. [18] reported phonatory function improvement after reconstruction of RLN in 88 patients with nerve resection and also in 51 (58%) patients who had UVFP preoperatively. Reconstruction of RLN may provide partial or full recovery from vocal fold atrophy and the returning thyroarytenoid muscle tone during phonation. 

Feasibility of the GRBAS scale for assessment of subjective voice outcome with laryngoplasty has been reported [11, 27, 28]. This study also indicated a G change from 1.2 to 0.2, an R change from 0.7 to 0.2, and a B change from 1.0 to 0.2 in group I patients with reinnervation RLN. In group II, mean values of G, R, and B *reached* the same values as Group I postoperatively. All G, R, and B scores were under 0.5 scales after the surgery. Perceptual analysis showed that our patients' voices were returned to normal or near normal after the surgery. 

 In this study, thyroid cancer was only limited to invasion of the unilateral RLN. There are some advanced invasions, such as to the trachea, larynx, or esophagus. In such advanced cases it may not be possible to reserve distal stumps of RLN at the entrance of the larynx. If there is no distal portion of the RLN left below the Berry's ligament, the inferior pharyngeal constrictor muscle should be divided along the lateral edge of the thyroid cartilage to find a distal stump of the RLN. The thyroid cartilage is retracted, and cricothyroid joint is opened. Behind the thyroid cartilage the RLN forms several branches. The abductor and adductor branches of the RLN are identified, and the adductor branch is dissected superior to ensure sufficient length for anastomosis. When adductor branches cannot be found and opposite RLN can be preserved, arytenoid adduction may be performed to keep phonatory function.

## 5. Conclusion

In this case series we reported favorable patient outcomes as measured by aerodynamic and perceptional analyses as well as videostroboscopic findings after immediate RLN reconstruction for severed RLN during thyroid cancer surgery. Also, we reported that our patients experienced a normal or improved voice postoperatively, regardless of the length of time they had suffered from UVFP. Despite these favorable results, the small sample size utilized in this study limits the conclusions that may be drawn. Further research could help to confirm the results and expand the application of this procedure.

The immediate RLN reconstruction for severed RLN during the thyroid cancer surgery is highly effective in preventing the loss of phonatory function.

## Figures and Tables

**Table 1:** List of twelve cases of immediate RLN reconstruction in the present series.

Patient No.	Age	Gender	Side	UVFP before surgery	Methods of reconstruction	Follow-up, mo
1	39	F	L	3 mo	FNG	27
2	64	F	L	2 mo	FNG	36
3	82	F	L	2 mo	FNG	12
4	69	F	R	—	FNG	103
5	35	F	L	13 mo	FNG	68
6	72	M	L	3 mo	FNG	18
7	74	F	L	—	ARA	7
8	71	F	R	3 mo	ARA	40
9	61	M	R	—	FNG	38
10	86	F	L	—	DA	36
11	18	M	R	—	FNG	22
12	71	M	R	—	FNG	9

UVFP: unilateral vocal fold paralysis, FNG: free nerve grafting, ARA: ansa cervicalis to RLN anastomosis, and DA: direct anastomosis.

**Table 2:** Preoperative and postoperative voice data.

	Patient No.	Mucosal wave		Glottal closure		MPT (sec)		MFR (ml/sec)		Grade		Roughness		Breathiness	
		Preop	Postop	Preop	Postop	Preop	Postop	Preop	Postope	Preope	Postop	Preop	Postop	Preop	Postop
Group I	1	1	3	1	3	8.9	26.5	184.0	86.0	1	0	0	0	1	0
	2	1	2	2	2	6.2	19.9	109.0	70.0	2	0	1	0	1	0
	3	3	3	2	3	9.6	13.5	116.0	104.0	0	0	0	0	0	0
	5	1	2	2	2	8.7	16.2	176.0	168.0	1	0	1	0	1	0
	6	0	2	0	3	2.7	10.6	932.0	146.0	2	1	2	1	2	1
	8	1	3	2	3	6.5	10.4	114.0	88.0	1	0	0	0	1	0
	Mean ± SD	1.2 ± 1.0	2.5 ± 0.5	1.5 ± 0.8	2.7 ± 0.5	7.1 ± 2.6	16.2 ± 6.2	271.8 ± 325	110.3 ± 38.4	1.2 ± 0.8	0.2 ± 0.4	0.7 ± 0.8	0.2 ± 0.4	1.0 ± 0.6	0.2 ± 0.4
Group II	4	3	2	3	3	22.2	25.4	61.0	62.0	0	0	0	0	0	0
	7	NA	3	NA	3	NA	14.9	NA	64.0	NA	0	NA	0	NA	0
	9	1	1	3	3	22.4	24.3	60.0	102.0	2	1	1	1	2	1
	10	IV	IV	IV	IV	11.3	11.5	41.0	78.0	0	0	0	0	0	0
	11	NA	2	NA	3	NA	12.0	NA	168.0	NA	0	NA	0	NA	0
	12	NA	3	NA	3	NA	17.7	NA	168.0	NA	0	NA	0	NA	0
	Mean ± SD	2.0± 1.4	2.2 ± 08	3.0 ± 0.0	3.0 ± 0.0	18.6 ± 6.4	17.6 ± 6.0	54.0 ± 11.3	107 ± 49.4	0.7 ± 1.2	0.2 ± 0.4	0.3 ± 0.6	0.2 ± 0.4	0.7 ± 1.2	0.2 ± 0.4

NA: not assessed, IV: invisible glottis during phonation, MPT: maximum phonation time, and MFR: mean airflow rate.

## References

[1] Hogikyan ND, Wodchis WP, Terrell JE, Bradford CR, Esclamado RM (2000). Voice-Related Quality of Life (V-RQOL) following type I thyroplasty for unilateral vocal fold paralysis. *Journal of Voice*.

[2] Spector BC, Netterville JL, Billante C, Clary J, Reinisch L, Smith TL (2001). Quality-of-life assessment in patients with unilateral vocal cord paralysis. *Otolaryngology*.

[3] Ford CN, Bless DM (1986). A preliminary study of injectable collagen in human vocal fold augmentation. *Otolaryngology*.

[4] Isshiki N, Okamura H, Ishikawa T (1975). Thyroplasty type I (lateral compression) for dysphonia due to vocal cord paralysis or atrophy. *Acta Oto-Laryngologica*.

[5] Isshiki N, Tanabe M, Sawada M (1978). Arytenoid adduction for unilateral vocal cord paralysis. *Archives of Otolaryngology*.

[6] Crumley RL (1991). Update: ansa cervicalis to recurrent laryngeal nerve anastomosis for unilateral laryngeal paralysis. *Laryngoscope*.

[7] Green DC, Berke GS, Graves MC (1991). A functional evaluation of ansa cervicalis nerve transfer for unilateral vocal cord paralysis: future directions for laryngeal reinnervation. *Otolaryngology*.

[8] Paniello RC (2004). Laryngeal reinnervation. *Otolaryngologic Clinics of North America*.

[9] Yumoto E, Sanuki T, Kumai Y (2006). Immediate recurrent laryngeal nerve reconstruction and vocal outcome. *Laryngoscope*.

[10] Lee WT, Milstein C, Hicks D, Akst LM, Esclamado RM (2007). Results of ansa to recurrent laryngeal nerve reinnervation. *Otolaryngology*.

[11] Hirano M (1981). Clinical examination of voice. *The Journal of the Acoustical Society of America*.

[12] Chhetri DK, Gerratt BR, Kreiman J, Berke GS (1999). Combined arytenoid adduction and laryngeal reinnervation in the treatment of vocal fold paralysis. *Laryngoscope*.

[13] Zheng H, Li Z, Zhou S, Cuan Y, Wen W (1996). Update: laryngeal reinnervation for unilateral vocal cord paralysis with the ansa cervicalis. *Laryngoscope*.

[14] Miyauchi A, Matsusaka K, Kihara M (1998). The role of ansa-to-recurrent-laryngeal nerve anastomosis in operations for thyroid cancer. *European Journal of Surgery*.

[15] Chiang F-Y, Wang L-F, Huang Y-F, Lee K-W, Kuo W-R (2005). Recurrent laryngeal nerve palsy after thyroidectomy with routine identification of the recurrent laryngeal nerve. *Surgery*.

[16] Miyauchi A, Yokozawa T, Kobayashi K, Hirai K, Matsuzuka F, Kuma K (2001). Opposite ansa cervicalis to recurrent laryngeal nerve anastomosis to restore phonation in patients with advanced thyroid cancer. *European Journal of Surgery*.

[17] Miyauchi A, Ito Y, Miya A (2007). Lateral mobilization of the recurrent laryngeal nerve to facilitate tracheal surgery in patients with thyroid cancer invading the trachea near Berry’s ligament. *World Journal of Surgery*.

[18] Miyauchi A, Inoue H, Tomoda C (2009). Improvement in phonation after reconstruction of the recurrent laryngeal nerve in patients with thyroid cancer invading the nerve. *Surgery*.

[19] Piñeros-Fernández A, Rodeheaver PF, Rodeheaver GT (2005). Octyl 2-cyanoacrylate for repair of peripheral nerve. *Annals of Plastic Surgery*.

[20] Landegren T, Risling M, Brage A, Persson JKE (2006). Long-term results of peripheral nerve repair: a comparison of nerve anastomosis with ethyl-cyanoacrylate and epineural sutures. *Scandinavian Journal of Plastic and Reconstructive Surgery and Hand Surgery*.

[21] Aubá C, Hontanilla B, Arcocha J, Gorría Ó (2006). Peripheral nerve regeneration through allografts compared with autografts in FK506-treated monkeys. *Journal of Neurosurgery*.

[22] Choi B-H, Kim B-Y, Huh J-Y (2004). Microneural anastomosis using cyanoacrylate adhesives. *International Journal of Oral and Maxillofacial Surgery*.

[23] Tseng YC, Hyon SH, Ikada Y, Shimizu Y, Tamura K, Hitomi S (1990). In vivo evaluation of 2-cyanoacrylates as surgical adhesives. *Journal of Applied Biomaterials*.

[24] Wieken K, Angioi-Duprez K, Lim A, Marchal L, Merle M (2003). Nerve anastomosis with glue: comparative histologic study of fibrin and cyanoacrylate glue. *Journal of Reconstructive Microsurgery*.

[25] Montanaro L, Arciola CR, Cenni E (2001). Cytotoxicity, blood compatibility and antimicrobial activity of two cyanoacrylate glues for surgical use. *Biomaterials*.

[26] Maronian N, Waugh P, Robinson L, Hillel A (2003). Electromyographic findings in recurrent laryngeal nerve reinnervation. *Annals of Otology, Rhinology and Laryngology*.

[27] Dulguerov P, Schweizer V, Caumel I, Esteve F (1999). Medialization laryngoplasty. *Otolaryngology*.

[28] Cummings CW, Purcell LL, Flint PW (1993). Hydroxylapatite laryngeal implants for medialization. Preliminary report. *Annals of Otology, Rhinology and Laryngology*.

